# Clinical characteristics and outcomes of children, adolescents and young adults with overweight or obesity and mental health disorders

**DOI:** 10.1038/s41366-023-01449-4

**Published:** 2024-01-09

**Authors:** Angela Galler, Angelika Thönnes, Jens Joas, Christine Joisten, Antje Körner, Thomas Reinehr, Markus Röbl, Gerd Schauerte, Wolfgang Siegfried, Daniel Weghuber, Susann Weihrauch-Blüher, Susanna Wiegand, Reinhard W. Holl, Nicole Prinz, APV Initiative

**Affiliations:** 1 Charité - Universitätsmedizin Berlin, corporate member of Freie Universität Berlin and Humboldt-Universität zu Berlin, Sozialpädiatrisches Zentrum, Paediatric Endocrinology and Diabetology, Berlin, Germany; 2 Universitätsklinikum des Saarlandes, Psychosomatische Medizin und Psychotherapie and Klinik für Kinder- und Jugendpsychiatrie, Psychosomatik und Psychotherapie, Homburg, Germany; 3 Universitätsklinikum des Saarlandes, Klinik für Kinder- und Jugendpsychiatrie, Psychosomatik und Psychotherapie, Homburg, Germany; 4 Deutsche Sporthochschule Köln, Institut für Bewegungs- und Neurowissenschaft, Köln, Germany; 5 Universitätsklinikum Leipzig, Klinik und Poliklinik für Kinder- und Jugendmedizin, Leipzig, Germany; 6 Vestische Kinderklinik Datteln, Datteln, Germany; 7 Universitätsmedizin Göttingen, Georg-August-Universität, Klinik für Kinder und Jugendmedizin, Göttingen, Germany; 8 CJD Berchtesgaden, Berchtesgaden, Germany; 9 Vorsorgeklinik INSULA/ Müttergenesungswerk, Bischofswiesen, Germany; 10 Universitätskinderklinik Salzburg, Salzburg, Austria; 11 Universitätsklinikum Halle/S., Dept. f. Pädiatrie I, Halle/Saale, Germany; 12 University of Ulm, Institute of Epidemiology and Medical Biometry, ZIBMT, Ulm, Germany; 13 German Center for Diabetes Research (DZD), Munich-Neuherberg, Germany

**Keywords:** Obesity, Risk factors, Paediatrics

## Abstract

**Background:**

Mental disorders are important comorbidities in youth with obesity. Aim was to describe the clinical characteristics and outcome of youth with overweight or obesity having comorbid mental disorders.

**Methods:**

Data from children, adolescents, and young adults (age 6–30 years) with overweight or obesity and mental disorders (depression, anxiety disorder, eating disorder, attention deficit disorder (ADHD)) from 226 centers in Germany and Austria participating in the Adiposity Patient Registry (APV) were analyzed and compared with those without reported mental disorders using regression modeling.

**Results:**

Mental health comorbidity was reported in a total of 3969 out of 114,248 individuals with overweight or obesity: 42.5% had ADHD, 31.3% anxiety disorders, 24.3% depression, and 12.9% eating disorders. Being male (OR 1.39 (95%CI 1.27;1.52)), of older age (1.42 (1.25;1.62)), or with extreme obesity (1.45 (1.30;1.63)) were most strongly associated with mental health comorbidity. Regression analysis showed that mean BMI-SDS was significantly higher in the group of individuals with depression and eating disorders (BMI-SDS 2.13 (lower; upper mean:2.09;2.16) and 2.22 (2.17;2.26)) compared to those without reported mental health comorbidity (BMI-SDS 2.008 (2.005;2.011); *p* < 0.001). In youth with ADHD, BMI-SDS was lower compared to those without reported mental disorders (BMI-SDS 1.91 (1.89;1.93) vs 2.008 (2.005;2.011); *p* < 0.001). Proportion of severe obesity was higher in individuals with depression (23.7%), anxiety disorders (17.8%), and eating disorders (33.3%), but lower in ADHD (10.3%), compared to those without reported mental disorders (13.5%, *p* < 0.002). Proportions of dyslipidaemia and abnormal carbohydrate metabolism were not different in youth with and without reported mental health comorbidity. BMI-SDS change after one year of lifestyle intervention program ranged between −0.22 and −0.16 and was similar in youth without and with different mental disorders.

**Conclusion:**

Health care professionals caring for youth with overweight or obesity should be aware of comorbid mental disorders and regular mental health screening should be considered.

## Introduction

The high prevalence of overweight and obesity in childhood, adolescence, and young adulthood is a global health problem. Prevalence estimates in Germany find that 15.4% of children and adolescents are overweight, including 5.9% affected by obesity [1]. Youth with overweight and obesity are more likely to suffer from health impairments such as hypertension, dyslipidemia, abnormal glucose metabolism, nonalcoholic fatty liver disease and orthopedic illnesses when compared to their normal weight peers [2–4]. Overweight and obesity in childhood and adolescence is related to higher risk of overweight in adulthood [5]. The consequences of childhood and adolescent obesity not only include health-related physical outcomes, but also impact social, behavioral, and emotional well-being. There is conclusive evidence, that children and adolescents with overweight or obesity have an increased risk of behavioral or emotional disorders like depression, eating disorders, and lower self-esteem [6, 7].

Generally, mental disorders affect a significant number of children and adolescents. According to a meta-analysis on the worldwide prevalence of mental disorders in children and adolescents, 13.4% meet the criteria for any mental disorders: 6.5% for anxiety disorders, 2.6% for major depressive disorder, 3.4% for attention deficit with/without hyperactivity disorder (ADHD), and 5.7% for any disruptive disorder [8]. Literature indicate an increasing burden of mental disorders [9]. Socioeconomic and functional aspects are important variables to consider in the development and prevention of psychological comorbidities in chronic somatic conditions, such as obesity in childhood and adolescence [10]. Depression, anxiety disorders, eating disorders, and ADHD are more prevalent in children and adolescents with obesity than in healthy weight youths [7]. For example, numerous studies report an association between depression and childhood obesity, such that adolescents with depression are at an increased risk for the onset and persistence of obesity during adolescence and young adulthood [6, 11–13]. Research also suggests that there is a reciprocal link between depression and obesity, such that obesity increases the risk for depression, but also depression predicts the development of obesity [13]. Moreover, some psychotropic medications, e.g. antidepressants, contribute to weight gain and thereby to obesity in children and adolescents [14]. Furthermore, there is evidence on the association between ADHD, a disorder marked by symptoms of inattention, impulsivity or hyperactivity, and obesity [15, 16]. The prevalence of ADHD is significantly higher among children with overweight or obesity (7%) compared to those with normal weight (3.5%) [17]. Finally, aberrant eating has been identified as a very important cause of obesity [18]. Binge eating disorder, defined in the Diagnostic and Statistical Manual of Mental Disorders (DSM) as consumption of an objectively large amount of food accompanied by a sense of loss of control over eating, is the most common form of disordered eating among individuals with obesity. Given the difficulties to quantify a large amount of food for growing children and adolescents, loss of control eating, defined as the subjective experience of amount of uncontrolled consumption, is utilized in lieu of binge eating to identify aberrant eating behavior in children [18]. Most studies reveal prevalence rates of binge eating disorder between 1% to 3% [19]. Children with loss of control eating or binge eating disorder are younger, more frequently have depressive symptoms, symptoms of anxiety, and lower self-esteem than those without binge eating, as well as a poorer outcome after family-based treatment [20].

This study focuses on some of the most common mental health comorbidities of childhood obesity namely depression, anxiety disorders, eating disorders, and ADHD. The objective was to compare clinical characteristics and outcomes between children, adolescents and young adults with overweight or obesity with and without a clinically recognized mental disorder who participated in a lifestyle intervention program.

## Methods

In this study, we used data from children, adolescents and young adults with overweight or obesity from the Adiposity Patient Registry (APV), a prospective standardized multicentre database used by inpatient and outpatient specialized centers for obesity care in Germany and Austria [21]. The APV initiative is open to all interested centers providing care for individuals living with overweight or obesity. University or community hospitals and medical centers, rehabilitation clinics, nutritional counseling practices, and pediatricians with focus on obesity care participate. A list of participating centers is attached in the Supplementary Appendix. At present, a total of 237 specialized obesity care centers offering obesity treatment programs engage in APV. In 2000, APV started with a small number of participating centers and within the first 10 years the numbers of centers increased continuously. Due to external factors (e.g. reimbursement of treatment programs) the numbers of participating centers varied slightly within the past 10 years. In Germany, participation in APV is mandatory for accreditation as obesity care center and sometimes also for cost reimbursement of treatment programs. Hence, most specialized obesity care centers take part in the APV initiative.

Each of the 237 obesity care centers records obesity-related data on all individuals at their center and electronically transfers anonymous data records biannually to the University of Ulm. Anthropometric, metabolic, psychosocial, and treatment-related parameters are collected via an electronic health record system. Demographics and basic obesity-related data (for example BMI, sex) are mandatory, but many other parameters are documented (e.g. blood pressure, laboratory data, comorbidities like mental disorders, and treatment modalities). The University of Ulm performs reliability checks and reports inconsistent data back to the centers for validation. Revision of data is done by each center and corrected anonymous data is sent back to University of Ulm. Informed consent was obtained and the Ethics Committee of Ulm University approved anonymized data collection and analysis (reference number 133/22).

We included data from 114,248 children, adolescents and young adults with overweight or obesity within the age range of 6 to ≤30 years from 226 centers participating in different lifestyle intervention programs within the time span from 2000 to 2021. We excluded individuals not participating in any lifestyle intervention program and individuals with syndromal obesity or individuals receiving bariatric surgery or using weight-modifying medications like metformin, orlistat or glucagon-like peptide-1 (GLP-1) analogs. Overall, 49,402 (43.2%) individuals participated in different lifestyle intervention programs in outpatient units (treatment modality: outpatient) and 64,846 (56.8%) individuals underwent different lifestyle intervention programs in rehabilitation centers including an inpatient stay in so-called rehabilitation units (treatment modality: inpatient). We searched the database for the diagnosis of mental disorders and the corresponding terms (e.g. F32.2 and/or major depressive disorder) according to the ICD-10 GM (International Statistical Classification of Diseases and Related Health Problems, 10th revision, German Modification) classification and to the Diagnostic and Statistical Manual of Mental Disorders – Fourth and Fifth Edition (DSM-IV and DSM-5). Individuals with mental disorders were categorized into the following mental disorder groups: Depression, anxiety or obsessive/compulsive disorders, eating disorders, and attention deficit disorder with/without hyperactivity (ADHD). For categorization into the groups depression or ADHD, we additionally searched for antidepressant or licensed ADHD medications, both trademark and generic nomenclature, because the use of antidepressant or ADHD medication is indicative of a diagnosis of depression or ADHD, respectively. Due to their overlapping symptoms, obsessive-compulsive disorders were included into the group of anxiety disorders. Binge eating disorders and loss of control over eating were included into the group eating disorders.

### Clinical and biochemical parameters

BMI was given as standard deviation score (SDS), using nationally representative reference values [22]. Overweight was defined as BMI ≥90th to <97th percentile, obesity as a BMI ≥97th percentile, and severe obesity as a BMI ≥ 99,5th percentile based on age and sex. BMI-SDS change after one year of lifestyle intervention program was calculated as the difference between the BMI-SDS at initial presentation and the aggregated BMI-SDS of the time period 0.5–1.5 years after the initial presentation. For the initial BMI-SDS, multiple values were aggregated over the first 21 days after starting a lifestyle intervention program. Migration background was defined if either the patient or at least one parent was born outside Germany, Austria, or Switzerland. For definition of ambulatory elevated blood pressure, we used the 95th age- and sex-specific percentile of the representative population based German study KIGGS [23]. Laboratory parameters were measured in local laboratories according to national guidelines [24]. According to the national guidelines, dyslipidaemia was assumed if total cholesterol levels were >200 mg/dl and/or HDL < 35 mg/dl and/or LDL > 130 mg/dl and/or triglycerides >150 mg/dl [25]. An abnormal carbohydrate metabolism was defined as either HbA1c ≥ 6.5% and or fasting blood glucose ≥100 mg/dl and/or 2-hour-glucose ≥140 mg/dl. HbA1c measurements were standardized to the Diabetes Control and Complication Trial (DCCT) reference range (4.05–6.05%; 20.7–42.6 mmol/mol) [26].

### Statistical analysis

Statistical evaluation was performed using SAS, version 9.4 (build TS1M7, SAS Institute Inc., Cary, NC, USA). Data are presented as median (first and third quartile) for continuous variables and as proportion for dichotomous parameters. Descriptive statistics were implemented for the whole study population and separately for the group of individuals with or without reported mental disorders and for each group of individuals with the different mental disorder categories. Of each individual, the most recent year of treatment was analyzed. In case of multiple datasets per individual, variables were aggregated as median. We analyzed differences in sex, age, migration background, initial BMI-SDS, obesity-related comorbidities, treatment modality, and BMI-SDS change after one year of follow-up in children, adolescents and young adults with vs without reported mental disorders and in those with different mental disorders. To explore age effects, individuals were divided into three age groups: 6- to 12-year-olds, 12- to 15-year-olds, and 15- to 30-year-olds. Continuous parameters like age were compared by Wilcoxon–Mann–Whitney-test. Proportions of migration background, elevated blood pressure, dyslipidemia, and abnormal carbohydrate metabolism were compared between the groups with different mental disorder categories and those without reported mental disorders (control group) by χ^2^-test. *P*-values were adjusted for multiple comparisons using the method of Bonferroni–Holm. Multivariable logistic regression was performed for the presence of any mental disorder to evaluate the association with different exploratory variables: age, sex, migration background, presence of severe obesity, dyslipidemia, elevated blood pressure, abnormal carbohydrate metabolism and treatment modality (outpatient/inpatient) and odds ratios (OR with 95%CI) were calculated. Separate multivariable linear and logistic regression models were conducted to examine the relationship between specific mental disorder categories and no reported mental disorders and the clinical variables BMI-SDS, proportion of severe obesity, dyslipidemia, abnormal carbohydrate metabolism, and BMI-SDS change after one year of follow-up, respectively. All models were adjusted for the following confounding variables: age (grouped), sex, and migratory background. The confounder age was categorized as 6–<12, 12–15, and >15–≤30 years. The models for dyslipidemia and abnormal glucose metabolism were additionally adjusted for initial BMI-SDS, and the model for BMI-SDS change was additionally adjusted for initial BMI-SDS and treatment modality, respectively. *P*-values were adjusted for multiple comparisons using the method of Tukey–Kramer. A two-sided probability value of less than 0.05 was considered significant.

## Results

**Fig. 1:**
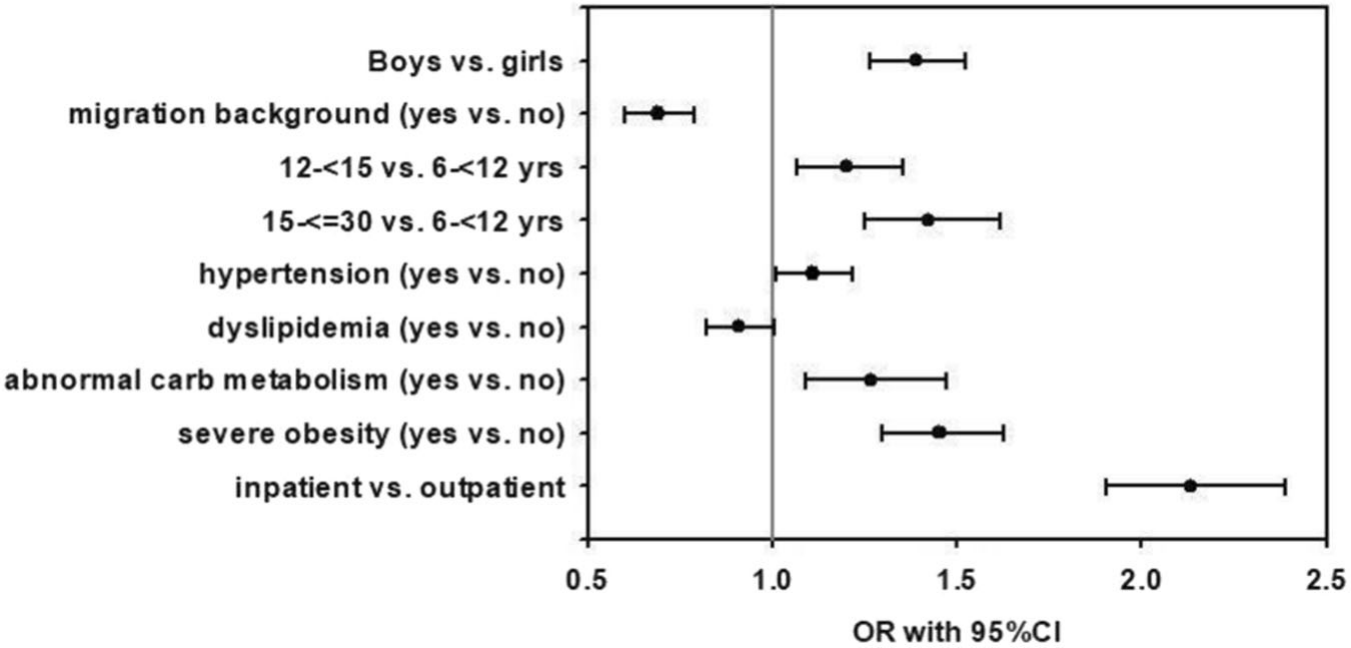
Odds ratios for the presence of mental disorders. Plot showing odds ratios (OR and 95%CI) for the presence of mental disorders in relationship to demographic and clinical risk factors in children, adolescents and young adults with overweight or obesity.

Characteristics of the whole study population are given in Table 1. Any comorbid mental disorder was reported in a total of 3969 children, adolescents and young adults with overweight or obesity. The group of youth with mental disorders comprised more males and older individuals, and migration background was less frequent compared to the group without reported mental disorders (see Table 1). We performed regression analysis in order to identify the factors associated with mental disorders in youth with overweight or obesity. Figure 1 depicts the odds ratios for the presence of mental disorders in relationship to demographic and clinical risk factors in children, adolescents and young adults with overweight or obesity. Being male (OR 1.39; 95% CI (1.27;1.52)), older age (1.42; (1.25;1.62)), and severe obesity (1.45; (1.30;1.63)) were most strongly associated with mental health comorbidity in youth with overweight or obesity (see Fig. 1). Furthermore, mental health comorbidity was strongly associated with a lifestyle intervention program in rehabilitation centers with an inpatient stay (see Fig. 1). Having a migration background was linked with lower odds of comorbid mental health comorbidity (see Fig. 1). Within the group of 3969 youths with mental disorders, 42.5% had ADHD, 31.3% had anxiety disorders, 24.3% had depression, and 12.9% had eating disorders. Out of the 3,969 individuals with mental health comorbidity, 88.1% had one mental disorder. In 10.4% of individuals, two comorbid mental disorders were recognized and 1.5% had three or more mental disorders.

**Table 1:** Characteristics of the study population.

	All	Without mental disorder	With mental disorder	${\rm{p}}$
Number	114,248	110,279	3969	–
Age (years)^a^	13.3 (11.3; 15.0)	13.2 (11.3; 15.0)	13.8 (12.2; 15.6)	<0.001^c^
Sex: male/female (%)^b^	47.0/53.0	46.8/53.2	53.7/46.3	<0.001^d^
Migration background (%)^b^	15.7	15.9	10.1	<0.001^d^
Age 6–>12 years (%)^b^	32.6	33.0	23.0	<0.001^d^
Age 12–<15 years (%)^b^	42.1	42.0	44.5	0.017^d^
Age 15–>30 years (%)^b^	25.3	25.0	32.5	<0.001^d^
Severe obesity (%)^b^	14.4	14.3	18.5	<0.001^d^
Initial BMI-SDS^a^	2.10 (1.81; 2.40)	2.10 (1.81; 2.40)	2.13 (1.82; 2.47)	<0.001^c^
BMI-SDS change after one year^a^	−0.14 (−0.32; −0.01)	−0,14 (−0.32; −0.01)	−0.20 (−0.43; −0.03)	<0.001^c^
Elevated blood pressure (%)^b^	54.6	54.7	53.1	0.39^d^
Dyslipidaemia (%)^b^	32.2	32.4	29.6	0.04^d^
HbA1c (%)^a^	5.3 (5.1; 5.5)	5.3 (5.1; 5.5)	5.3 (5.1; 5.5)	1.0^c^
Abnormal carbohydrate metabolism (%)^b^	10.5	10.5	11.5	0.72^d^
Treatment modality: inpatient/outpatient (%)^b^	56.8/43.2	56.1/43.9	75.1/24.9	<0.001^d^

^a^Median (first quartile; third quartile).

^b^Proportion.

^c^Wilcoxon–Mann–Whitney-test for comparison of individuals with vs without mental disorder.

^d^χ²-test for comparison of individuals with vs without mental disorder.

Characteristics of the children, adolescents and young adults with different mental disorders (depression, anxiety disorder, eating disorder, or ADHD) are depicted in Table 2. In Table 2, individuals with more than one mental disorder were categorized into the larger group. We observed several differences with respect to sex, age, proportion of severe obesity, and treatment modality between the different mental disorder categories (see Table 2): The groups of youth with depression and eating disorders comprise more females and older individuals compared to the individuals without reported mental disorders or those with ADHD. Moreover, severe obesity was more prevalent in the groups of individuals with depression, anxiety disorders, and eating disorders (26.8%, 18.4%, and 37.4%, respectively), whereas it was less prevalent in the ADHD group (10.9%) compared to the group without reported mental health comorbidity (14.3%, see Table 2).

**Table 2:** Characteristics of children, adolescents and young adults with overweight or obesity without and with different mental disorders.

	Without mental disorder	Depression	*p*	Anxiety disorder	*p*	Eating disorder	*p*	ADHD^a^	*p*
Number	110,279	810	–	1131	–	340	–	1688	–
Age (years)^b^	13.2 (11.3; 15.0)	15.0 (13.5; 16.9)	<0.001^d^	13.3 (11.6; 15.1)	0.42^d^	15.1 (13.3; 17.2)	<0.001^d^	13.3 (11.8; 14.90)	0.07^d^
Sex: male/female (%)^c^	46.8/53.2	33.7/66.3	<0.001^e^	45.8/54.2	1.0^e^	37.1/62.9	0.005^e^	72.0/28.0	<0.001^e^
Migration background (%)^c^	15.9	9.0	<0.001^e^	10.3	<0.001^e^	14.4	1.0^e^	9.7	<0.001^e^
Age 6->12 years (%)^c^	33.0	10.3	<0.001^e^	30.2	0.71^e^	10.0	<0.001^e^	26.8	<0.001^e^
Age 12-<15 years (%)^c^	42.0	39.3	0.70^e^	43.1	1.0^e^	37.9	1.0^e^	49.4	<0.001^e^
Age 15->30 years (%)^c^	25.0	50.5	<0.001^e^	26.7	1.0^e^	52.1	<0.001^e^	23.8	1.0^e^
Severe obesity (%)^c^	14.3	26.8	<0.001^e^	18.4	0.002^e^	37.4	<0.001^e^	10.9	0.001^e^
Initial BMI-SDS^b^	2.10 (1.81; 2.40)	2.29 (1.96; 2.61)	<0.001^d^	2.15 (1.84; 2.45)	0.02^d^	2.43 (2.05; 2.76)	<0.001^d^	2.02 (1.75; 2.33)	<0.001^d^
BMI-SDS change after one year^b^	−0.14 (−0.32; −0,01)	−0.26 (−0.49; −0.07)	<0.001^d^	−0.21 (−0.40; −0.03)	0.01^d^	−0.36 (−0.64; −0.01)	<0.001^d^	−0.17 (−0.36; −0.01)	0.78^d^
Elevated blood pressure (%)^c^	54.7	47.2	<0.001^e^	53.4	1.0^e^	57.1	1.0^e^	55.1	1.0^e^
Dyslipidaemia (%)^c^	32.4	31.3	1.0^e^	27.4	0.09^e^	30.8	1.0^e^	30.0	0.78^e^
HbA1c (%)^b^	5.3 (5.1; 5.5)	5.3 (5.0; 5.6)	1.0^d^	5.3 (5.1; 5.4)	1.0^d^	5.2 (5.0; 5.4)	1.0^d^	5.3 (5.1; 5.5)	1.0^d^
Abnormal carbohydrate metabolism (%)^c^	10.5	10.9	1.0^e^	9.4	1.0^e^	12.5	1.0^e^	13.1	0.18^e^
Treatment modality: inpatient/outpatient (%)^c^	56.1/43.9	86.3/13.7	<0.001^e^	74.1/25.9	<0.001^e^	80.3/19.7	<0.001^e^	69.3/30.7	<0.001^e^

^a^Attention deficit disorder with/without hyperactivity.

^b^Median (first quartile; third quartile).

^c^Proportion.

^d^Wilcoxon–Mann–Whitney-test for comparison of individuals with vs without mental disorder, *p*-values adjusted for multiple comparisons using the method of Bonferroni Holm.

^e^χ²-test for comparison of individuals with vs without mental disorder, *p*-values adjusted for multiple comparisons using the method of Bonferroni–Holm.

Because of the significant differences with respect to sex, age, and migration background among the groups with the different mental disorders, we performed regression analysis adjusting for sex, age, and migration background. Comparisons of BMI-SDS and percentage of severe obesity in youth with overweight or obesity among the different comorbid mental disorder categories are depicted in Table 3A: Mean BMI-SDS was significantly higher in the group of individuals with depression and eating disorders compared to those without reported mental health comorbidity. In contrast, mean BMI SDS was not different between individuals with anxiety disorder and without reported mental disorders. In youth with ADHD, mean BMI-SDS was lower compared to those individuals without reported mental disorders (see Table 3A). Mean BMI-SDS in the group with ADHD was lowest, whereas mean BMI-SDS was highest in the group with eating disorders (see Table 3A). Severe obesity was most present in the group with eating disorders, followed by the group with depression and anxiety disorders. The group of ADHD significantly comprised less individuals with severe obesity (see Table 3A). Regression analysis comparing the proportion of dyslipidaemia, adjusted for sex, age, migration background and initial BMI-SDS, revealed that there were slightly but significantly less individuals with dyslipidaemia in the group with anxiety disorders whereas there were no differences between the other mental disorder categories (see Table 3B). We found no differences with respect to the proportion of abnormal carbohydrate metabolism (see Table 3B). Lastly, we performed regression analysis, adjusted for sex, age, migration background, initial BMI-SDS, and treatment modality in order to compare BMI-SDS change after one year of follow-up between the different groups. Adjusted estimated mean BMI-SDS change after one year of lifestyle intervention ranged between −0.22 and −0.16 and was not different between the groups of individuals with different mental disorders and those without reported mental health comorbidity (see Table 4).

**Table 3:** **A**. Comparison of BMI SDS and proportion of extreme obesity in children, adolescents, and young adults with overweight or obesity without and with different mental disorders, adjusted for sex, age, and migration background; **B**. Comparison of proportion of dyslipidaemia and abnormal carbohydrate metabolism in children, adolescents, and young adults with overweight or obesity without and with different mental disorders, adjusted for sex, age, migration background, and initial BMI-SDS.




Table A. Upper half: Comparison of estimated mean BMI-SDS by linear regression model, adjusting for sex, age, and migration background with ∆ = difference in BMI-SDS and SE = standard error, *p*-values adjusted for multiple comparisons using the method of Tukey–Kramer.

Lower half: Comparison of estimated mean proportion of severe obesity by logistic regression model, adjusting for sex, age, and migration background with ∆ = difference in proportion of extreme obesity (%), *p*-values adjusted for multiple comparisons using the method of Tukey–Kramer.

^a^Attention deficit disorder with/without hyperactivity.

^b^Estimated mean BMI-SDS (lower mean; upper mean).

^c^Estimated mean proportion of severe obesity (%) (lower mean; upper mean).

Table B. Upper half: Comparison of estimated mean proportion of dyslipidaemia by logistic regression model, adjusting for sex, age, migration background, and initial BMI-SDS with ∆ = difference in proportion of dyslipidaemia (%), *p*-values adjusted for multiple comparisons using the method of Tukey–Kramer

Lower half: Comparison of estimated mean proportion of abnormal carbohydrate metabolism by logistic regression model, adjusting for sex, age, migration background, and initial BMI-SDS with ∆ = difference in proportion of abnormal carbohydrate metabolism (%), *p*-values adjusted for multiple comparisons using the method of Tukey–Kramer.

^a^Attention deficit disorder with/without hyperactivity.

^b^Estimated mean proportion of dyslipidaemia (%) (lower mean; upper mean).

^c^Estimated mean proportion of abnormal carbohydrate metabolism (%) (lower mean; upper mean).

**Table 4:** Comparison of BMI-SDS change after one year of lifestyle intervention program in children, adolescents, and young adults with overweight or obesity without and with different mental disorders, adjusted for sex, age, migration background, initial BMI-SDS, and treatment modality.

Group (*N* = 27,580)	Without mental disorder	Depression	Anxiety disorder	Eating disorder	ADHD^a^
BMI-SDS change^b^	−0.187 (−0.191; −0.184)^b^	−0.16 (−0.21; −0.10)^b^	−0.22 (−0.25; −0.18)^b^	−0.22 (−0.28; −0.16)^b^	−0.19 (−0.22; −0.16)^b^
Without mental disorder −0.187 (−0.191; −0.184)^b^	–	∆ = 0.03 SE = 0.026 *p* = 0.75^c^	∆ = 0.03 SE = 0.019 *p* = 0.58^c^	∆ = 0.03 SE = 0.030 *p* = 0.83^c^	∆ = 0.004 SE = 0.015 *p* = 1.0^c^
Depression −0.16 (−0.21; −0.10)^b^	∆ = 0.03 SE = 0.026 *p* = 0.75^c^	–	∆ = 0.06 SE = 0.032 *p* = 0.34^c^	∆ = 0.06 SE = 0.039 *p* = 0.49^c^	∆ = 0.04 SE = 0.030 *p* = 0.76^c^
Anxiety disorder −0.22 (−0.25; −0.18)^b^	∆ = 0.03 SE = 0.019 *p* = 0.58^c^	∆ = 0.06 SE = 0.032 *p* = 0.34^c^	–	∆ = 0.004 SE = 0.035 *p* = 1.0^c^	∆ = 0.02 SE = 0.024 *p* = 0.85^c^
Eating disorder −0.22 (−0.28; −0.16)^b^	∆ = 0.03 SE = 0.030 *p* = 0.83^c^	∆ = 0.06 SE = 0.039 *p* = 0.49^c^	∆ = 0.004 SE = 0.035 *p* = 1.0^c^	–	∆ = 0.03 SE = 0.033 *p* = 0.92^c^
ADHD^a^ −0.19 (−0.22; −0.16)^b^	∆ = 0.004 SE = 0.015 *p* = 1.0^c^	∆ = 0.04 SE = 0.030 *p* = 0.76^c^	∆ = 0.02 SE = 0.024 *p* = 0.85^c^	∆ = 0.03 SE = 0.033 *p* = 0.92^c^	–

^a^Attention deficit disorder with/without hyperactivity.

^b^Estimated mean BMI-SDS change (lower mean; upper mean).

^c^Comparison of estimated mean BMI-SDS change after one year of lifestyle intervention program by linear regression model, adjusting for sex, age, migration background, initial BMI-SDS, and treatment modality with ∆ = difference in BMI-SDS change and SE = standard error, *p*-values adjusted for multiple comparisons using the method of Tukey–Kramer.

## Discussion

This is a study aiming to describe clinical characteristics and outcome of a large number of children, adolescents and young adults with overweight or obesity with mental health comorbidity. We found noticeable differences between youths with or without reported mental disorders: Being male, of older age and affected by severe obesity were most strongly associated with mental health comorbidity in children, adolescents and young adults with overweight or obesity. However, looking at demographic factors in more detail, we found that the majority of individuals with depression and eating disorders were females whereas there were more males than females with ADHD. BMI-SDS was higher in youth with depression and eating disorders compared to those with anxiety disorder, ADHD, or without reported mental disorder. In individuals with depression, anxiety disorder, and eating disorder, severe obesity was more frequent compared to those with ADHD or those without reported mental health comorbidity. Proportions of comorbidities like dyslipidaemia and abnormal carbohydrate metabolism were not different in youth with depression, eating disorder, ADHD, and without reported mental health comorbidity. BMI-SDS change after one year of lifestyle intervention program was not different between individuals with different mental disorders and those without reported mental health comorbidity.

So far, only few studies have evaluated the impact of mental health comorbidities on clinical characteristics and outcome of youth with overweight or obesity. As expected, age and sex were associated with different mental disorders in our study, e.g. we found more females and older individuals with depression and more males and younger individuals with ADHD. This fits the general observation, that prevalence of depression is increasing with age in childhood, adolescence and young adulthood, and more females are affected. Moreover, it is well known that ADHD is diagnosed and treated more often in males than in females [27]. In our study, the most frequent reported mental disorder was ADHD. At first glance this seems surprising, because in the general population anxiety disorders are more common than ADHD [8]. Though, several studies found that anxiety disorders are often unrecognized and underdiagnosed in children, adolescents and young adults [28, 29]. In contrast, ADHD is probably suspected and diagnosed more often by clinicians because impulsive and hyperactive behavior is presumably more conspicuous than other emotional symptoms and results in further diagnostic testing. Overall, migration background was less represented in youths with mental disorders in our study. Although the prevalence of mental disorders in children and adolescents with migration background varies by ethnic origin of the immigrant, country of immigration, time since immigration, and also by first- or second-generation immigration status, many studies find a lower prevalence of behavioral problems and mental disorders in children and adolescents with migration background [30]. Furthermore, persons with migration background usually attend mental health care services less frequently than the autochthonous population [31]. The reasons for these differences possibly relate to deficits in utilization of mental health care services and to a different understanding of dealing with psychological stress in the migrant population.

Consistent with many studies, we found higher BMI-SDS in youth with depression and eating disorders compared to those without reported comorbid mental disorders [6, 7, 11–13, 18, 19]. Moreover, more females with obesity are diagnosed with eating disorders. Many studies and a meta-analysis report on the association between depressive symptoms or depression and obesity in childhood and adolescence [6, 11–13, 31–33]. There is also clear data from numerous studies and a meta-analysis about the relationship between eating disorders and obesity [6, 18–20]. Hence, our results are very well in line with international data. In our study, BMI-SDS was not higher in individuals with anxiety disorders and even lower in youth with ADHD compared to those without reported mental health comorbidities. The association between anxiety disorders or ADHD and obesity in the published literature is less clear compared to the data regarding depression and eating disorders [6]. It remains unclear as to whether emotional problems are a cause or a consequence of childhood obesity, probably common factors promote both [6]. Studies evaluating the relationship between obesity and anxiety disorders demonstrate controversial results [6]. Although youth with severe obesity had higher anxiety scores, youth with overweight or milder obesity did not consistently have more anxiety symptoms [6, 33]. This could explain our observation that mean BMI-SDS in the group of individuals with anxiety was not as high as in the group with depression and eating disorders but similar to the group without reported mental disorders. For ADHD and obesity, the results from international studies are mixed [6]: There is evidence on the association between ADHD and overweight or obesity [6, 15, 16]. The strength of association varies across research studies and also by gender. A representative study by Waring and Lapane [34] shows that after adjustment for age, gender, ethnicity, socioeconomic status, and depression/anxiety, children and adolescents with ADHD not currently using medication were about 1.5 times more likely to be overweight as compared to children and adolescents without ADHD. Moreover, children and adolescents with ADHD were about 9 times more likely to report depression or anxiety [34]. Additionally, analysis of a cross-sectional, nationally representative German sample of 2,863 parents and their children aged 11–17 years reveals associations between overweight and ADHD [17]. Results indicate that the prevalence of ADHD was significantly higher among children with overweight or obesity (7%) compared to those with normal weight (3.5%) [17]. However, other studies do not find any associations between ADHD and obesity [6]. For example, in a nationally representative and longitudinal study from Ireland, the authors find a co-occurrence of ADHD and obesity which is mainly explained by several psychosocial factors [35]. In our analysis, mean BMI-SDS and the proportion of severe obesity in the ADHD group were lower compared to the group of youth with other mental comorbidities and those without reported mental disorders, supporting the hypothesis that the association between obesity and ADHD is not very strong compared to other mental disorders.

Interestingly, proportions of the comorbidities dyslipidaemia and abnormal carbohydrate metabolism were not different in most groups with mental disorders compared to those without reported mental health comorbidity in our analysis. In contrast, adults with depression show an increased risk for developing pathological glucose metabolism and type 2 diabetes as demonstrated in numerous studies [36]. Possibly, children, adolescents and young adults with their shorter duration of overweight or obesity have not yet developed metabolic consequences. Interestingly, we found similar success rates regarding BMI change after one year of lifestyle intervention programs in youth with and without mental disorders. Generally, treatment of childhood obesity is based on behavioral lifestyle modifications. Lifestyle intervention programs have shown modest effect on weight loss, particularly in children with severe obesity, but aim to prevent or delay the progression of physiological comorbidities and show positive effect on the quality of life [37–39]. In clinical practice, lifestyle interventions are able to reduce BMI by 0.05–0.42 BMI-SDS over 1–24 months [37, 38]. BMI-SDS change after one year of lifestyle intervention program in our study ranged between −0.22 and −0.16 and thus lied within the expected range. The presence of a high social risk burden (for example low education, migration background, low parental employment) as a negative predictor for successful weight loss is well documented [40]. There is limited information on the impact of mental disorders on the efficacy of lifestyle intervention programs among children, adolescents, and young adults with overweight and obesity but one would probably expect that mental disorders have a negative impact on weight loss. Therefore, it was surprising, that BMI change was similar after one year of lifestyle intervention programs in youth with and without reported mental disorders. There are several possible explanations for this. Individuals with mental disorders probably benefit in a similar way from the structured lifestyle intervention program than individuals without mental disorders. Probably, these programs also positively influence the pathogenic patterns of depression and eating disorders through activation, structuring and psychological support, in particular in individuals with mental disorder, and thus could explain the weight loss success and outcome in all youth. In adults with obesity undergoing bariatric surgery an alike effect is seen: A meta-analysis evaluating mental health comorbidities and outcome after bariatric surgery shows that neither depression nor binge eating disorders are consistently associated with outcomes with respect to weight [41]. The authors found no clear evidence that preoperative mental health conditions are associated with differential weight loss after bariatric surgery. Thus, individuals with mental disorders have similar benefits and treatment success like individuals without mental health comorbidity. Consistent with that, we demonstrated similar BMI-SDS changes after one year of lifestyle intervention program in youth with or without reported mental comorbidities. Another explanation might be that individuals with mental disorders had auxiliary treatment, e.g. cognitive behavioral therapy or other psychotherapy, in addition to the regular lifestyle intervention program. For example, treatment in rehabilitation centers with an inpatient stay (which normally comprises a more extensive lifestyle intervention program) was more frequent in individuals with mental disorders compared to those without mental comorbidities in our study. This could add to the success in weight loss. Psychotherapy is especially important in eating disorders because among the children receiving lifestyle intervention, those with aberrant eating behavior (for example binge eating) show an increase in the percentage weight gained at the end of the intervention as compared to children without eating disorders [20]. Unfortunately, we did not have any information about additional treatments or therapies. Lastly, there are likely more individuals with (undiagnosed) mental disorders in the group of individuals without reported mental disorders. These individuals presumably did not get further diagnostic testing, therefore probably less additional support and treatment, resulting in less weight loss success.

There are several strengths in the present study. First, we report on a large number of individuals with overweight or obesity with a clinically recognized comorbid mental disorder. Second, to our knowledge, there is scarce data about the presence of mental disorders and somatic comorbidities in children, adolescents and young adults. Most of the studies focus either on only somatic consequences and risks, for example pathological glucose metabolism or dyslipidaemia, or on only behavioral or emotional symptoms and effects. In our study we examine associations between different mental disorders and somatic consequences. There are also several limitations. First, the data reported comes from a registry in which obesity centers caring for children, adolescents and young adults with overweight or obesity participate. Therefore, the reported data of these individuals is not representative for all children, adolescents and young adults with overweight or obesity in Germany and Austria. Hence, the findings have limited generalizability. For example, the rates of severe obesity or comorbidities (e.g. mental disorders) might be higher in individuals being cared for in obesity centers in this study compared to individuals not treated in specialized centers. Second, we only examined associations and we are not able to draw any conclusions with respect to cause or effect between the factors obesity and mental disorders. Furthermore, we did not have any information about socioeconomic status, about additional psychological interventions for the children, adolescents and young adults with or without mental health comorbidities. Though, these factors are well known to have some effect on treatment outcome. Lastly, there is very likely some underreporting of mental disorders in the group of youth without reported mental health comorbidities. In our study, only the mental disorders, which were clinically recognized and reported in clinical everyday practice, are included and no additional psychological testing was done for this analysis.

## Conclusions

Health care professionals should be aware of mental health comorbidity in children, adolescents and young adults with overweight or obesity. In youth with depression or eating disorders BMI-SDS is higher and severe obesity is more frequently prevalent whereas the prevalence rates of dyslipidaemia and abnormal glucose metabolism seem similar in youth with or without mental disorders. Interdisciplinary lifestyle intervention programs consisting of dietary interventions, physical activity programs, and psychological and medical care seem to benefit children and young adolescents with overweight or obesity both with or without mental disorders.

### Supplementary information

Appendix

## Data Availability

The datasets generated during and analyzed during the current study are available from the corresponding authors on reasonable request.

## References

[1] Schienkiewitz A, Brettschneider AK, Damerow S, Rosario AS (2018). Overweight and obesity among children and adolescents in Germany. Results of the cross-sectional KiGGS Wave 2 study and trends. J Health Monit.

[2] Kiess W. Risikofaktoren der Adipositas im Kindes- und Jugendalter. In: Herpertz S, Zwaan M, Zipfel S, editors. Handbuch Essstörungen und Adipositas. 3rd ed. Berlin, Heidelberg: Springer; 2022. pp 481–5.

[3] Bohn B, Wiegand S, Kiess W, Reinehr T, Stachow R, Oepen J (2017). APV Initiative and the German Competence Network Obesity. Changing Characteristics of Obese Children and Adolescents Entering Pediatric Lifestyle Intervention Programs in Germany over the Last 11 Years: An Adiposity Patients Registry Multicenter Analysis of 65,453 Children and Adolescents. Obes Facts.

[4] Koutny F, Weghuber D, Bollow E, Greber-Platzer S, Hartmann K, Körner A (2020). Prevalence of prediabetes and type 2 diabetes in children with obesity and increased transaminases in European German-speaking countries. Analysis of the APV initiative. Pediatr Obes.

[5] Singh AS, Mulder C, Twisk JW, van Mechelen W, Chinapaw MJ (2008). Tracking of childhood overweight into adulthood: a systematic review of the literature. Obes Rev.

[6] Rankin J, Matthews L, Cobley S, Han A, Sanders R, Wiltshire HD (2016). Psychological consequences of childhood obesity: psychiatric comorbidity and prevention. Adolesc Health Med Ther.

[7] Kalarchian MA, Marcus MD (2012). Psychiatric comorbidity of childhood obesity. Int Rev Psychiatry.

[8] Polanczyk GV, Salum GA, Sugaya LS, Caye A, Rohde LA (2015). Annual research review: a meta-analysis of the worldwide prevalence of mental disorders in children and adolescents. J Child Psychol Psychiatry.

[9] Steffen A, Akmatov MK, Holstiege J, Bätzing J. Diagnoseprävalenz psychischer Störungen bei Kindern und Jugendlichen in Deutschland: eine Analyse bundesweiter vertragsärztlicher Abrechnungsdaten der Jahre 2009 bis 2017. Zentralinstitut für die kassenärztliche Versorgung in Deutschland (Zi). Versorgungsatlas-Bericht Nr. 18/07. Berlin. 2018; 10.20364/VA-18.07.

[10] Erhart M, Weimann A, Bullinger M, Schulte-Markwort M, Ravens-Sieberer U (2011). Psychische Komorbidität bei chronisch somatischen Erkrankungen im Kindes-und Jugendalter. Bundesgesundheitsblatt-Gesundheitsforschung-Gesundheitsschutz.

[11] Goodman E, Whitaker RC (2002). A prospective study of the role of depression in the development and persistence of adolescent obesity. Pediatrics.

[12] Hasler G, Pine DS, Kleinbaum DG, Gamma A, Luckenbaugh D, Ajdacic V (2005). Depressive symptoms during childhood and adult obesity: the Zurich Cohort Study. Mol Psychiatry.

[13] Luppino FS, Wi LM, de Bouvy PF, Stijnen T, Cuijpers P, Penninx BW (2010). Overweight, obesity, and depression: a systematic review and meta-analysis of longitudinal studies. Arch Gen Psychiatry.

[14] Reekie J, Hosking SPM, Prakash C, Kao KT, Juonala M, Sabin MA (2015). Antidepressants/psychotics and weight in youth. Obes Rev.

[15] de Zwaan M, Gruß B, Müller A, Philipsen A, Graap H, Martin A (2011). Association between obesity and adult attention-deficit/hyperactivity disorder in a German community-based sample. Obesity Facts.

[16] Cortese S, Moreira-Maia CR, St. Fleur D, Morcillo-Peñalver C, Rohde LA, Faraone SV (2016). Association between ADHD and obesity: a systematic review and meta-analysis. Am J Psychiatry.

[17] Erhart M, Herpertz-Dahlmann B, Wille N, Sawitzky-Rose B, Hölling H, Ravens-Sieberer U (2012). Examining the relationship between attention-deficit/hyperactivity disorder and overweight in children and adolescents. Eur Child Adolesc Psychiatry.

[18] Marcus MD, Kalarchian M (2003). Binge eating in children and adolescents. Int J Eat Disord.

[19] Wolkoff LE, Tanofsky-Kraff M, Shomaker LB, Kozlosky M, Columbo KM, Elliott CA (2011). Self-reported vs actual energy intake in youth with and without loss of control eating. Eating Behav.

[20] Wildes JE, Marcus MD, Kalarchian MA, Levine MD, Houck PR, Cheng Y (2010). Self-reported binge eating in severe pediatric obesity: impact on weight change in a randomized controlled trial of family-based treatment. Int J Obes.

[21] Prinz N, Pomares-Millan H, Dannemann A, Giordano GN, Joisten C, Körner A (2023). Who benefits most from outpatient lifestyle intervention? An IMI-SOPHIA study on pediatric individuals living with overweight and obesity. Obesity.

[22] Schaffrath Rosario A, Kurth BM, Stolzenberg H, Ellert U, Neuhauser H (2010). Body mass index percentiles for children and adolescents in Germany based on a nationally representative sample (KiGGS 2003–2006). Eur J Clin Nutr.

[23] Neuhauser HK, Thamm M, Ellert U, Hense HW, Rosario AS (2011). Blood pressure percentiles by age and height from nonoverweight children and adolescents in Germany. Pediatrics.

[24] Nauck M (2019). Golfier A: Laboratoriumsmedizinische Untersuchungen: Richtlinie zur Qualitätssicherung der Bundesärztekammer (BÄK) überarbeitet. Dtsch Arztebl.

[25] Thierfelder W, Dortschy R, Hintzpeter B, Kahl H, Scheidt-Nave C (2007). Biochemische Messparameter im Kinder- und Jugendgesundheitssurvey (KiGGS). Bundesgesundheitsbl.

[26] Rosenbauer J, Dost A, Karges B, Hungele A, Stahl A, Bächle C (2012). Improved metabolic control in children and adolescents with type 1 diabetes: a trend analysis using prospective multicenter data from Germany and Austria. Diabetes Care.

[27] Biederman J, Mick E, Faraone SV, Braaten E, Doyle A, Spencer T (2002). Influence of gender on attention deficit hyperactivity disorder in children referred to a psychiatric clinic. Am J Psychiatry.

[28] Essau CA, Conradt J, Petermann F (2000). Frequency, comorbidity, and psychosocial impairment of anxiety disorders in German adolescents. J Anxiety Disord.

[29] Hansen BH, Oerbeck B, Skirbekk B, Kristensen H (2016). Non-obsessive–compulsive anxiety disorders in child and adolescent mental health services – Are they underdiagnosed, and how accurate is referral information?. Nord J Psychiatry.

[30] Singh GK, Yu SM, Kogan MD (2013). Health, chronic conditions, and behavioral risk disparities among U.S. immigrant children and adolescents. Public Health Rep.

[31] Quek YH, Tam WWS, Zhang MWB, Ho RCM (2017). Exploring the association between childhood and adolescent obesity and depression: a meta-analysis. Obes Rev.

[32] Sutaria S, Devakumar D, Yasuda SS, Das S, Saxena S (2019). Is obesity associated with depression in children? Systematic review and meta-analysis. Arch Dis Child.

[33] Lindberg L, Hagman E, Danielsson P, Marcus C, Persson M (2020). Anxiety and depression in children and adolescents with obesity: a nationwide study in Sweden. BMC Med.

[34] Waring ME, Lapane KL (2008). Overweight in children and adolescents in relation to attention-deficit/hyperactivity disorder: results from a national sample. Pediatrics.

[35] Phillips BA, Gaudette S, McCracken A, Razzaq S, Sutton K, Speed L (2012). Psychosocial functioning in children and adolescents with extreme obesity. J Clin Psychol Med Settings.

[36] Yu M, Zhang X, Lu F, Fang L (2015). Depression and risk for diabetes: a meta-analysis. Can J Diabetes.

[37] Reinehr T, Schaefer A, Winkel K, Finne E, Toschke AM, Kolip P (2010). An effective lifestyle intervention in overweight children: findings from a randomized controlled trial on “Obeldickslight”. Clin Nutr.

[38] Mühlig Y, Wabitsch M, Moss A, Hebebrand J (2014). Weight loss in children and adolescents. Dtsch Arztebl Int.

[39] Mollerup PM, Nielsen TRH, Bøjsøe C, Kloppenborg JT, Baker JL, Holm JC (2017). Quality of life improves in children and adolescents during a community-based overweight and obesity treatment. Qual Life Res.

[40] Koplan JP, Liverman CT, Kraak VI, Committee on Prevention of Obesity in Children and Youth. (2005). Preventing childhood obesity: health in the balance: executive summary. J Am Diet Assoc.

[41] Dawes AJ, Maggard-Gibbons M, Maher AR, Booth MJ, Miake-Lye I, Beroes M (2016). Mental health conditions among patients seeking and undergoing bariatric surgery: a meta-analysis. JAMA.

