# Supplementary material for: Clinical characteristics and outcomes of children, adolescents and young adults with overweight or obesity and mental health disorders
Source: Int J Obes (Lond). 2024 Jan 9;48(3):423–32. doi: 10.1038/s41366-023-01449-4 (PMC10896720; doi:10.1038/s41366-023-01449-4)
Supplement: Supplementary file 1 — Appendix [file 41366_2023_1449_MOESM1_ESM.docx]

**Appendix**

Participating centres in the APV initiative:

Amrum Satteldüne Kinder-Reha, Augsburg Bunter Kreis, Bad Bodenteich Moby Kids Seeparkklinik, Bad Fallingbostel Gesundheitszentrum, Bad Frankenhausen Kinder-Reha, Bad Heilbrunn Praxis für Ernährungsberatung, Bad Hersfeld Kinderklinik, Bad Kreuznach Viktoriastift, Bad Kösen Kinder-Reha, Bad Lippspringe, Bad Mergentheim Kinderklinik, Bad Neuenahr - DRK signatursambulanz, Bad Orb Spessartklinik - Kinder-Reha, Bad Rothenfelde Kinder-Reha, Bad Salzungen Reha-Klinik Charlottenhall, Bad Segeberg ZIMT, Bensheim Ernährungspraxis, Berchtesgaden CJD, Berchtesgaden Klinik Schönsicht Kinder-Reha, Berghaupten TOP-LIFE, Berlin Charite Kinderklinik, Berlin DRK Moby Kids, Berlin Lichtenberg Kinderklinik, Berlin Pfundskinder, Berlin Vivantes Beh.Zentrum SPZ, Berliner Jugendrotkreuz (JRK), Bischofswiesen/Strub, INSULA, Blaubeuren Ernährungspraxis, Bonn Ernährungsberatung KIDS Schulung, Bonn Universitäts-Kinderklinik, Braunschweig ernährungsmed. Zentrum, Bregenz - Landeskrankenhaus Kinderklinik, Bremen – ZABS, Bremen Zentralkrankenhaus Kinderklinik, Bremen-Nord Kinderklinik, Bruchweiler Kinder-Reha, Brügge, Fördekids, Buchholz Ernährungsberatung, Böblingen Kinderarztpraxis, Bühl - Praxis Ernährungsberatung, Cottbus - Reha Vita, Darmstadt Kinderklinik, Datteln Vestische Kinderklinik, Delmenhorst Kinderklinik, Detmold Kinderklinik, Dieburg Ernährungsberatung KIDS Schulung, Dinslaken Kinderklinik, Dornbirn Kinderklinik, Dorsten St. Elisabethkrhs., Dresden Moby Kids, Düren Gesundheitsamt, Düren sozialpäd. Zentrum Marienhospital, Düsseldorf Ernährungsberatung "richtig essen", Düsseldorf Ernährungspraxis "iss gut", Eppingen Kinderarztpraxis Schulze, Erlangen Uni-Kinderklinik, Eschede Adipositastraining KIDS, Essen Kinder und Jugendpsychiatrie, Ettenheim Kinderarztpraxis, Euskirchen Kinderarztpraxis, Feldberg Fachklinik Caritas-Haus, Feldkirch Landeskrankenhaus Kinderklinik, Flensburg Fördekids, Frankfurt Päd. Endokrinologie, Freiburg – Fitoc, Freiburg Uni-Kinderklinik Freinsheim Ernährungsberatung, Friedrichsdorf Ernährungsberatung, Friedrichsdorf Ernährungspraxis, Fulda Ernährungsberatung Osthessen, Fürth Kinderklinik, Gaissach Fachklinik Deutsche Rentenversicherung Bayern-Süd, Garz Fachklinik CJD, Gauting, Kinderarztpraxis, Gelnhausen Ernährungsberatung, Gelsenkirchen Bergmannsheil Kinderklinik Buer, Gera SRH Wald-Klinikum, Gera Waldklinikum, Gittelde am Harz – Ernährungsberatung, Gotha Helios Kinderklinik, Greifswald Neuropädiatrie/Stoffwechsel, Göttingen Uni-Kinderklinik, Göttingen interdis. Adipositaszentrum, Göttingen, KIDS Schulungsprogramm, Hagen Allgemeines Krankenhaus, Hagen Kinderarztpraxis, Hagen Kinderklinik, Halle Universitäts-Kinderklinik, Hamburg Moby Kids, Hamburg Moby Kids Partner Konopka, Hamburg Rallye Energy, Hamburg Wilhelmstift, Hamburg-Sprungbrett, Hannover BKK Essanelle, Hannover Kinderklinik Bult, Haßfurt Adipositasschulung Haßberge, Hemer Ernährungspraxis Schweizer, Herdecke Kinderklinik, Herne Praxis Ernährungsmedizin, Herrenberg JumboKids, Hilden Hausarztpraxis, Hirschberg Praxis Maurer, Homburg CJD, Homburg Uni-Kinderklinik, Kassel Kinderarztpraxis, Kiel städt. Krankenhaus Fördekids, Korbach Ernährungsberatung, Krefeld Kinderklinik, Kreischa Klinikum Bavaria Zscheckwitz, Kronshagen Praxis, Köln - Amsterdamerstrasse, Power Pänz, Köln - Prävention UniReha GmbH, Köln MeLo KIDS Schulungsprogramm, Köln Sporthochschule, Köln endlichVital | SI Ernährungssignatur, Kölpinsee, Seebad Klaus Störtebecker Kinder-Reha, Lahr Praxis für Ernährung, Leipzig - KLAKS e. V., Leipzig Uni-Kinderklinik, Leverkusen Kinderklinik, Lindau Forum Adipositas e.V., Lindenberg/Lindau Adipositasschulung, Lingen Bonifatius-Hospital, Luhe Ernährungsberatung/LuheVitalConcept, Lörrach Kinderklinik, Lübeck Uni-Kinderklinik, Magdeburg - Städtische Kinderklinik, Magdeburg Uni-Kinderklinik, Magdeburg VSB 1980 - bärenstark abnehmen, Mahlow Programm TRI FIT junior, Menden BIG, Munster Ernährungs- & Bewegungsschulung für K&J, Murnau Kinder-Reha, Mönchengladbach Städt. Kinderklinik, Mühlhausen Präventionspraxis Scherf, München Adieupositas, München Barmherzige Brüder Innere Med, München Ernährungssignatur Kinderleicht, Münster ADI MOBIL, Nettetal eat Ernaehrungsberatung, Neumünster Präventionszentrum, Neunkirchen Kinderklinik, Neuss Lukaskrankenhaus, Niederkassel Kinderarztpraxis Sprenker, Norden - Klinik Nordendeich, Nürnberg PEP, Nürnberg Praxis Landendörfer, Oberhausen Adipositaszentrum, Oberhausen EKO Kinderklinik, Oberstaufen Ernährungsmedizin, Oberstenfeld Ernährungspraxis, Oberstenfeld Ernährungspraxis2, Oldenburg Kids-Schulungsprogramm, Oldendorf Ernährungspraxis KiloKids, Osnabrück christliches Kinderhospital, Overath KIDS-Schulungsprogramm, Oy-Mittelberg Reha, Paderborn Ernährungspraxis, Passau Kinderklinik, Pforzheim Adipositas Training, Pforzheim Ernährungsberatung eat&move, Pleidelsheim Adipositas-Zentrum Bietigheim-Bissingen, Pocking Kinderarztpraxis, Poppenricht Ernährungsberatung, Potsdam Patienten Trainings Zentrum, Pönitz FiFaFu KIDS-Programm, Ravensburg Ernährung und Diät, Ravensburg Oberschwabenklinik Kinderklinik, Regensburg Kinderarztpraxis, Reiskirchen Ernährungspraxis, Rendsburg Villa Schwensen, Praxisgemeinschaft KJPP, Rickert Ernährungsberatung, Ronneburg Ernährungsberatung, Rosenheim Lufti-Team, Rottweil Kinder-Leicht, Rüsselsheim Gesundheits- und Pflegezentrum, Saalfeld Kinderklinik, Saarbrücken Moby Kids, Salzburg Kinderklinik, Salzburg Uni-Kinderklinik, Salzgitter Kinderklinik, Scheidegg Prinzregent Luitpold Reha, Schliengen Ernährungsberatung, Schliengen Ernährungstherapie, Schliengen Erwachsene, Schrobenhausen Kreiskrankenhaus ZIZ4KIDS, Seebad Heringsdorf - Kinder-Reha, Senden Ernährungsberatung, Siegburg KIDS Schulungsprogramm, Siegen DRK Kinderklinik, Simonswald Klinik Eichhof, Solingen Ernährungsberatung, Sonneberg KIDS Ernährungspraxis, St. Augustin Kinderklinik, St. Pölten Landesklinikum Kinderklinik, Straubing Praxis, Tholey / SPZ Neunkirchen, Tübingen Universitäts-Kinderklinik, Ulm Uni-Kinderklinik, Untergruppenbach Ernährungsberatung, Vechta Praxis, Viersen Kinderklinik Nikolaus, Villingen Kinder-leicht-Programm, Waldbröl Gemeinschaftspraxis, Waltrop Ernährungsberatung, Wangen Kinder-Rehaklinik, Weilheim Kinder- und Jugendärzte, Weißendorf Praxis Ernährung Gesundheit, Wesseling Dreifaltigkeitskrankenhaus, Westerland/Sylt, Haus Quickborn, Westerland/Sylt, Kinder-Reha, Wien Ernährungsakademie, Wien Uni-Kinderklinik, Wiesbaden DKD Kinderklinik, Wiesmoor KIDS Schulungsprogramm, Windach Psychosomatik - Sportverein Triathlon, Witten Kinderarztpraxis, Wuppertal Helios Kinderklinik, Wustrow Ostseebad Fischland, Wyk auf Föhr - AOK Kinderkurheim, Würzburg ambulantes Schulungszentrum, Zorneding Ernährungsberatung, Zwickau - Praxis Ernährungsberatung
